# Pattern and Frequency of Congenital Heart Defects Among Infants of Diabetic Mothers

**DOI:** 10.7759/cureus.76184

**Published:** 2024-12-22

**Authors:** Hussain A Al Ghadeer, Ahmed H Alherz, Fatimah S Albattat, Mohammed A Alkhamis, Mohammed H Alamer, Luay F Almulaifi, Ali I Al Ali, Nazihah A Al Nowaiser, Zakariya S Aldandan, Abdullah H Al khamis, Ali N Bumejdad, Ameen A Alali, Zainab a aljubarah, Abdullah H Almeshari, Mohammed A AlJumaah

**Affiliations:** 1 Paediatrics, Maternity and Children Hospital, AlAhsa, SAU; 2 Neonatology, Maternity and Children Hospital, AlAhsa, SAU; 3 Paediatrics, Oyun City Hospital, AlAhsa, SAU

**Keywords:** alahsa, cardiac defects, congenital heart diseases, diabetic mothers, echocardiography, gestational diabetes mellitus, pre-gestational diabetes mellitus, saudi arabia

## Abstract

Background

Maternal diabetes mellitus (DM) is a known risk factor for congenital heart diseases (CHDs), which are of significant concern to infants born to diabetic mothers. Compared to newborns born to non-diabetic mothers, infants born to diabetic mothers had a higher overall risk of developing congenital malformations. This association has a complex pathophysiology that includes genetic predispositions, metabolic abnormalities, and environmental factors during key stages of fetal development. By developing screening strategies for neonates born to diabetic mothers, it will be imperative to reduce preventable neonatal mortality by healthcare providers.

Purpose

The primary objective of this study was to explore the spectrum of congenital heart defects (infants of diabetic mothers, IDMs).

Methods

This exploratory study was conducted at the maternity and children's hospital in AlAhsa, Saudi Arabia, from 2022 to 2023. The study included 401 neonates delivered at our institution. Within the first seven days of life, an expert pediatric cardiologist from the same institute performed echocardiography on all patients.

Results

A total of 401 infants born to diabetic mothers were selected, with 293 meeting the inclusion criteria. In total, 144 (49.1%) were boys and 149 (50.9%) were girls. Nearly more than half of mothers (189, 64.5%) had gestational diabetes, while 104 (35.5%) had pre-gestational diabetes. Out of 293 infants born to diabetic mothers, 200 (68.3%) had various CHDs, while the remaining 93 (31.7%) were found to be normal after echocardiography. The most commonly reported CHD is patent ductus arteriosus (PDA) (71.5%), followed by hypertrophic cardiomyopathy (36.5%) and ventricular septal defect (VSD) (11%), and only one patient has a complex congenital heart disease.

Conclusion

More than half of the infants born to diabetic mothers had congenital heart defects, according to the current study, which examined the various types of congenital heart diseases in neonates of diabetic mothers. It thus emphasizes the necessity of a thorough evaluation and the strong recommendation for an early diagnosis of CHD in this high-risk group. In our population, prenatal CHD screening programs need to be developed.

## Introduction

Diabetes mellitus (DM) is a metabolic disorder characterized by hyperglycemia, which can be caused by either a decrease or absence of insulin secretion, increased resistance, or both. DM is considered a major health issue worldwide [1]. In 2019, a study estimated the global diabetes prevalence: 463 million patients have DM. In 2030, it is expected to rise to 578 million patients with DM, and in 2045, it will rise up to 700 million [2]. The World Health Organization (WHO) reported that Saudi Arabia ranks second in the Middle East and seventh globally in terms of the high prevalence of DM, with 14.4% of the population suffering from the disease (14.7% of males and 13.8% of females) and 5% of deaths due to DM [3]. At least 10% of all pregnancies are affected by DM, which makes it a common complication of pregnancy [4]. DM during pregnancy is classified as (i) pregestational diabetes mellitus (PDM) type 1 (T1DM) or type 2 (T2DM) and (ii) gestational diabetes mellitus (GDM). GDM occurs during pregnancy [5]. Ninety percent of DM during pregnancy is GDM, while pre-existing diabetes is 10%, with a gradual increase worldwide [4,6]. Risk factors of DM are advanced maternal age, obesity, family history, sedentary lifestyle, unhealthy diet, and smoking [7].

DM has an adverse effect on both fetal and maternal outcomes. Hyperglycemia during critical periods in pregnancy is a primary driver of adverse outcomes. Outcomes of DM pregnancy depend on the level of maternal glucose and the time of uncontrolled DM during pregnancy. Uncontrolled DM early in pregnancy during organogenesis is associated with a high risk of congenital anomalies, macrosomia, abortion, birth asphyxia, and preterm delivery [8,9]. Congenital cardiovascular defects are among the most common types of birth defects, accounting for approximately seven to eight babies per 1000 live births. The cause of cardiovascular defects is usually unknown; however, some are hereditary, and a small number are environmental. Maternal illness accounts for approximately one out of every hundred of these cases [10]. It was observed that a decrease in the neonatal mortality rate was brought about by an increase in pregnancy terminations and post-cardiac surgery survival [11]. Congenital defects and anomalies are more common in infants born to mothers with diabetes. Compared to normal neonates, the overall risk of developing congenital malformations increases by 2-12% in infants born to diabetic mothers [12].

Therefore, it is important to estimate the burden of diabetes and its complications on fetal and maternal health to improve the outcomes for these high-risk pregnancies. The primary objective of this study was to explore the spectrum of cardiovascular complications in infants of diabetic mothers (IDMs).

Research significance/importance

Prepregnancy care for women with pregestational type 1 or type 2 diabetes mellitus is effective in improving rates of perinatal morbidity and mortality and in reducing maternal HbA1C. Many mothers with diabetes are prone to recurrent pregnancy loss, stillbirth, and multiple congenital anomalies when they become pregnant. This study will provide baseline data on pregestational and gestational diabetes mellitus in this setting, serving as a valuable reference for future studies in this field. This study will help plan for applicable methods to improve fetal and maternal outcomes.

## Materials and methods

Study design and population

It was a cross-sectional study conducted at Maternity and Children Hospital, AlAHsa, Saudi Arabia, over two years, from January 2022 to December 2023. Infants born to diabetic mothers (T1DM, T2DM, and gestational DM) were enrolled in the study, and the frequency and pattern of congenital heart diseases (CHDs) were determined by echocardiography. The mothers who have gestational and pregestational DM and gave birth to a neonate with a gestational age of 24 weeks or more were included in the study. Infants of mothers who had multiple comorbidities other than DM were excluded.

Data collection

The data were collected from January 2022 to December 2023 from patients who delivered at the Maternity and Children's Hospital, AlAhsa, through the medical records. The fetal cardiac outcomes were obtained from the cardiology department through the echocardiography report and follow-up.

The collected data were divided into three parts. The first part includes sociodemographic data of the mother, such as age, history of miscarriage, or neonatal death. The second part includes information about the mother's history of diabetes mellitus, including the type of the disease, the type of treatment, the duration of the treatment, and her HbA1C. The third part covers the fetal outcomes, birth weight, and cardiac outcomes.

Data analysis

The data were collected, reviewed, and then analyzed by Statistical Package for Social Sciences version 21 (SPSS: IBM Corp., Armonk, NY). All statistical methods were two-tailed with an alpha level of 0.05, considering significance if the P-value is less than or equal to 0.05. Descriptive analysis was done by prescribing frequency distribution and percentage for participants' bio-demographic data, obstetric history, neonatal death, maternal outcome, cardiac outcome, and management received. The overall prevalence of cardiac outcome was graphed. Cross-tabulation was used to assess factors associated with the cardiac outcome of infants for diabetic mothers using Pearson chi-square and exact probability tests (for small frequency distribution). A multiple stepwise logistic regression model was used to assess the most significant adjusted predictors of cardiac outcome based on the backward regression method using the adjusted odds ratio with its 95% confidence interval.

## Results

A total of 293 eligible infants were included. Maternal age ranged from 18 to more than 40 years, with a mean age of 32.4 ± 11.9 years old (Table 1). Approximately 98 (33.4%) diabetic mothers had a history of neonatal death, mainly due to unknown causes due to either recall bias or not knowing the case of death after investigation (62.2%). The most known causes of neonatal death are uncontrolled DM (11.2%), congenital anomalies (9.2%), congenital heart diseases (6.1%), and prematurity (4.1%). As for the index child gender, 149 (50.9%) were females.

**Table 1:** Bio-demographic characteristics data of diabetic mothers, Al-Ahsa, Saudi Arabia (n=293)

Socio-demographic data	No	%
Maternal age		
18-30	95	32.4%
30-40	146	49.8%
>40	52	17.7%
History of neonatal death		
Yes	98	33.4%
No	195	66.6%
If yes, what is the cause?		
Unknown	61	62.2%
Uncontrolled DM	11	11.2%
Congenital anomalies	9	9.2%
Congenital heart disease	6	6.1%
Prematurity	4	4.1%
Neurological anomalies	2	2.0%
Vascular abnormality	2	2.0%
Ectopic pregnancy	1	1.0%
Molar pregnancy	1	1.0%
Ruptured membrane	1	1.0%
Child gender		
Male	144	49.1%
Female	149	50.9%

Diabetes data and treatment among study mothers (Table 2). Exactly 189 (64.5%) of the study mothers had gestational DM, 73 (24.9%) had type II DM, and 31 (10.6%) had type I DM. Considering the duration of diabetes, 117 (40.1%) had GDM for the first time, and 72 (24.7%) had previous GDM. Considering other types, 61 (20.9%) were diabetic for more than five years but 11 (3.8%) for less than one year. A total of 128 (43.7%) diabetic mothers were on diet control, 97 (33.1%) were on insulin, 48 (16.4%) were on medications, and 20 (6.8%) had a combined treatment regimen. One hundred and twenty-nine (44%) mothers had uncontrolled diabetes during pregnancy, while 102 (34.8%) received preconception care.

**Table 2:** Diabetes data and management among mothers, Al-Ahsa, Saudi Arabia (n=293)

Diabetes data	No	%
Type of DM		
DM type 1	31	10.6%
DM type 2	73	24.9%
Gestational DM	189	64.5%
Gestational DM		
1st GDM	117	40.1%
Previous GDM	72	24.7%
Duration of pregestational DM		
<1 year	11	3.8%
1–5 years	31	10.6%
>5 years	61	20.9%
Treatment regimen		
Diet	128	43.7%
Medication	48	16.4%
Insulin	97	33.1%
Combined	20	6.8%
Controlling of DM during pregnancy		
Controlled	164	56.0%
Uncontrolled	129	44.0%
Preconception care		
Yes	102	34.8%
No	191	65.2%

A total of 216 (37.7%) of the study infants born to diabetic mothers had a significant cardiac abnormality (Table 3). Cardiac auscultation showed that 124 (42.3%) infants had a murmur, ECG showed that 68 (23.2%) infants had prolonged QT interval, ECHO showed that 116 (39.6%) had isolated CHD, 84 (28.7%) had multiple CHD, and 58 (19.8%) mothers had infants with significant hemodynamics. With regard to management, most infants (71.8%) needed only medical observation, 55 (25.5%) needed medical treatment, and only 6 (2.7%) needed medical and surgical intervention.

**Table 3:** Cardiac outcomes among infants of diabetic mothers, Al-Ahsa, Saudi Arabia (n=293) Hemodynamic significant*: that affects the clinical condition of the patient and requires immediate medical or surgical intervention

Cardiac outcome	No	%
Auscultation		
Murmur	124	42.3%
Normal	169	57.7%
ECG		
Prolonged QT interval	68	23.2%
Normal	225	76.8%
Cardiac outcomes (ECHO finding)		
Isolated CHD	116	39.6%
Multiple CHD	84	28.7%
Normal	93	31.7%
Hemodynamic significant*		
Yes	58	19.8%
No	235	80.2%
Management (n=216)		
Observation	155	71.8%
Medical	55	25.5%
Medical and surgical	6	2.7%

Table 4 examines the types of congenital heart diseases among infants of diabetic mothers. The most commonly reported cyanotic congenital heart diseases are PDA (71.5%) and ventricular septal defect (11%). On the other hand, tetralogy of Fallot (TOF) (2.5%) was the most common among cyanotics. The second most commonly reported CHD following PDA is hypertrophic cardiomyopathy (36.5%).

**Table 4:** Type of congenital heart diseases among infants of diabetic mothers, Al-Ahsa, Saudi Arabia (n=293)

Cardiac outcome	No	%
Hypertrophic cardiomyopathy	73	36.5
Mild	50	25
Moderate	14	7
Severe	9	4.5
Acyanotic congenital heart disease	182	91
Patent ductus arteriosus (PDA)	143	71.5
Ventricular septal defect (VSD)	22	11
Small	17	8.5
Moderate	4	2
Large	1	0.5
Atrial septal defect (ASD) Type II	17	8.5
Cyanotic congenital heart disease	6	3
Tetralogy of Fallot (TOF)	5	2.5
Ebstein anomaly	1	0.5
Valvular heart disease	13	6.5
Tricuspid regurgitation	1	0.5
Mitral regurgitation	4	2
Pulmonary stenosis	5	2.5
Bicuspid aortic valve	3	1.5
Others	10	5
Persistent pulmonary hypertension (PPHN)	9	4.5
Complex congenital heart disease	1	0.5

Table 5 presents the factors linked to the cardiac outcome of infants born to diabetic mothers. Exact 80.6% of diabetic mothers with a history of neonatal death had infants with a significant cardiac outcome versus 70.3% of others without (P=0.048). Also, low birth weight infants had a significant cardiac outcome compared to normal birth weight infants (P=0.042). Significant cardiac outcome was detected among 87.1% of infants for mothers with DM for one to five years versus 45.5% of those with diabetes for less than one year (P=0.049).

**Table 5:** Factors associated with the cardiac outcome of infants for diabetic mothers P: Pearson X^2^ test ^Exact probability test *P < 0.05 (significant)

Factors	Significant cardiac finding				p-value
	Yes		No		
	No	%	No	%	
Maternal age					0.835^
<18	2	100.0%	0	0.0%	
18–30	69	74.2%	24	25.8%	
30–40	106	72.6%	40	27.4%	
>40	39	75.0%	13	25.0%	
Child gender					0.270
Male	102	70.8%	42	29.2%	
Female	114	76.5%	35	23.5%	
History of neonatal death					0.048*
Yes	79	80.6%	19	19.4%	
No	137	70.3%	58	29.7%	
Gestational age					0.891^
Very preterm (32–28 weeks)	1	100.0%	0	0.0%	
Moderate preterm (32 to <34 weeks)	2	66.7%	1	33.3%	
Late preterm (34 to <37 weeks)	48	71.6%	19	28.4%	
Full term (37–42 weeks)	165	74.3%	57	25.7%	
Birth weight					0.042*^
VLBW (<1500–1000 g)	2	100.0%	0	0.0%	
LBW (<2500–1500 g)	22	62.9%	13	37.1%	
NBW (2500–3999 g)	157	72.4%	60	27.6%	
HBW (≥4000 g)	35	89.7%	4	10.3%	
Type of DM					0.485
DM type 1	24	77.4%	7	22.6%	
DM type 2	57	78.1%	16	21.9%	
Gestational DM	135	71.4%	54	28.6%	
Gestational DM					0.049*
1st GDM	82	70.1%	35	29.9%	
Previous GDM	53	73.6%	19	26.4%	
Duration of pregestational DM					
<1 year	5	45.5%	6	54.5%	
1-5 years	27	87.1%	4	12.9%	
>5 years	48	78.7%	13	21.3%	
Treatment regimen					0.313
Diet	88	68.8%	40	31.3%	
Medication	39	81.3%	9	18.8%	
Insulin	73	75.3%	24	24.7%	
Combined	16	80.0%	4	20.0%	
Controlling of DM					0.611
Controlled	119	72.6%	45	27.4%	
Uncontrolled	97	75.2%	32	24.8%	
Preconception care					0.957
Yes	75	73.5%	27	26.5%	
No	141	73.8%	50	26.2%	

Multiple stepwise logistic regression for predictors of cardiac outcome among infants of diabetic mothers (Table 6). Among all included factors, the table included factors that were the most significant predictors of having a cardiac outcome, adjusting for all other factors. History of neonatal death increased the likelihood of having infants with cardiac outcomes by 89% (OR=1.89; 95% CI: 1.04-3.44). Also, increased duration of DM increased the risk by 11% (OR=1.11; 95% CI: 1.01-3.03), and infants with low birth weight had 82% more risk for having cardiac anomalies (OR=1.82; 95% CI: 1.09-3.22), keeping all other factors constant.

**Table 6:** Multiple stepwise logistic regression for predictors of cardiac outcome among infants of diabetic mothers AOR: adjusted odds ratio; CI: confidence interval; *P < 0.05 (significant)

Predictors	p-value	AOR	95% CI	
			Lower	Upper
History of neonatal death	0.036	1.89	1.04	3.44
Duration of DM	0.150	1.11	1.01	3.03
Low birth weight	0.020	1.82	1.09	3.22

## Discussion

The current study aimed to assess the incidence of cardiac outcomes in infants of diabetic mothers in maternity and children's hospitals in Al-Ahsa, Saudi Arabia. Pregnancy-related impaired glucose tolerance varies in frequency from 3% to 10%, depending on the average prevalence of diabetes in the general population [13,14]. Maternal DM targets the fetal heart through a complex pathogenesis, affecting both structure and function. The severity of damage is linked to DM type, early pregnancy HbA1c level, hyperglycemia, and hyperketonaemia, which causes toxicity to the developing embryo, increases apoptosis, and impairs cell homeostasis [15,16]. The current study showed that most mothers were above 30 years old and had high gravidity and parity. Also, the most prevalent diabetes was gestational DM, while other types were also reported. The dietary regimen was the most used among study mothers, with less than half of them having controlled diabetes during pregnancy and about one-third receiving preconception care.

As for the pregnancy outcome, the study showed that most infants were delivered by CS at full term with normal birth weight. Only one-fourth of the infants of the diabetic mothers were preterm, and less percent had low birth weight. A study conducted in King Khalid Hospital revealed that 21.6% of infants for diabetic mothers were delivered by CS, which is much less than the reported incidence in the current study [17]. Other adverse outcomes include large birth weight infants, congenital malformations, intrauterine growth retardation, and other severe forms of morbidity often associated with maternal diabetes status [18-21].

Regarding cardiac outcome, the current study revealed that about three-fourths of the infants of diabetic mothers had a significant cardiac outcome(s). Most murmurs were reported by auscultation, prolonged QT intervals were detected among about one-fourth of them, and CHDs were detected among two-thirds of the infants by ECHO. The most significant determinants of cardiac outcomes were a history of neonatal deaths, a long duration of diabetes, and low birth weight. Similarly, Øyen et al. [22] reported that a four-time increase in the infants' risk of CHDs was associated with maternal diabetes. Similar findings were also reported in the United States [23], Norway [24], and, lately, Canada [25]. In contrast to the current study, other studies revealed that gestational DM was only weakly associated with an increased risk of CHD [26]. Literature also revealed that among the most prevalent malformations in IDMs, cardiovascular abnormalities occur in 3% to 9% of diabetes pregnancies and are 2.5-10 times more likely than in normal pregnancies. If a mother has gestational diabetes and becomes insulin resistant in the third trimester, her relative risk of serious cardiovascular problems is highest [27]. When the mother's HbA1c level was less than 8.5, the reported incidence of problems was 3.4%, and when it was greater than 8.5%, it was 22.4%, which is all less than the reported incidence in the current study infants. Neonatal problems are more common in babies born to women who had an HbA1c level above 10% throughout their late pregnancy [28]. In roughly 25% to 30% of cases, fetal cardiac hypertrophy is documented as a consequence of pregestational or prenatal maternal diabetes [29]. Similar to the current study, it found that birth weight is the best predictor of hypertrophied IVS, especially in infants born to suboptimally controlled diabetic mothers [30]. This condition may be asymptomatic but may present with respiratory distress, cardiomegaly, signs of poor cardiac output, or even frank heart failure in about 5-10% of cases.

The literature revealed that DM, with its hyperglycemic situation before and during the early months of pregnancy, is associated with diabetic complications in the developing fetus, as it affects the heart, the massive vessels, and the neural tube [31]. Also, improper diabetes management and insufficient prenatal care are important confounding variables that raise the risk. Todorova et al. [32] found strong evidence linking elevated levels of glycated hemoglobin to fetal malformations, particularly during the first trimester of pregnancy. Additionally, they discovered that there was a higher likelihood of fetal cardiac disease in pregnancies with inadequate first-trimester glycemic control [33]. Research has shown that women with adequate glycemic control in a normal range during the time of conception and the early stages of pregnancy, as well as close monitoring, have a significantly lower risk of having a child with congestive heart failure than women with inadequate control. On its own, maternal smoking during pregnancy may raise the risk of CHD. Furthermore, pregestational diabetes and maternal tobacco use exacerbate each other's effects on preterm birth and the chance of congenital abnormalities, such as CHD [34].

With regard to types of congenital heart diseases, the most reported among the current study infants were PDA (most cases), but other anomalies were also reported, such as mild hypertrophic cardiomyopathy (one-fourth of the cases), with much lower frequency anomalies, including ASD II, small VSD, moderate hypertrophic cardiomyopathy, and PPHN. The literature showed that the most common incident cardiac anomalies in IDMs were ventricular septal defect, transposition of major arteries, and aortic stenosis. Defects involving the great arteries, including the truncus arteriosus and double outlet right ventricle, are also more prevalent in IDMs [35,36]. According to Vural et al. [37], symptomatic hypertrophic cardiomyopathy (HC) affects 12.1% of IDM patients; an echocardiographic scan routinely detects it in 30%, a finding that aligns with the current study frequency. Another study by Pabis et al. [38] reported a high frequency of HC (up to 40%) among infants of diabetic mothers due to hyperinsulinemia. Other studies assessed pulmonary hypertension [27,39,40]. In Saudi Arabia, Alyousif et al. [41] found that the most common congenital heart diseases in infants of diabetic mothers were patent ductus arteriosus (PDA) (31.7%), patent foramen ovale (PFO) (31.7%), and atrial septal defect (ASD) (23.6%). Abu-Sulaiman and Subaih [42] conducted a different Saudi study in which they found that 70% of IDMs had PDA, 68% had PFO, 5% had ASD, 4% had VSD, 2% had mitral valve prolapse, and 1% had pulmonary stenosis. A different study revealed that asymmetrical septal hypertrophy, PFO, and PDA were the most prevalent echocardiographic abnormalities in IDMs, accounting for 80%, 37.5%, and 27.5% of cases, respectively [43].

This study has certain limitations, including the absence of follow-up for IDMs regarding their echocardiographic findings, as well as the investigation of IDMs with significant congenital heart diseases and their relationship to HbA1c. Also, the current study is cross-sectional. Furthermore, the study did not examine the potential impact of glycemic management during pregnancy on the prevalence of congenital abnormalities in the fetus. There was only one center where the research was done. Multicenter research conducted in various Saudi Arabian regions would offer a more thorough understanding of the relationship between maternal diabetes and CHDs.

## Conclusions

In conclusion, the current study assesses the high incidence of cardiac outcomes among infants of diabetic mothers, mainly those with long-duration DM, mothers with a history of neonatal death, and infants born with low birth weight. Maternal DM significantly affects the fetal heart and placental circulation. Most cases needed only medical observation, with very few cases needing surgical intervention. Continuous assessment with proper prenatal and postnatal care for mothers with diabetes and their infants is mandatory to avoid life-threatening complications, including cardiac anomalies.
